# ID 105 - Tomografia por Emissão de Pósitrons Combinada à Tomografia Computadorizada (PET-CT) para Estadiamento de pacientes com Câncer de Esôfago: revisão sistemática e metaanálise

**DOI:** 10.5327/2237-9622.2025.v34s1.105

**Published:** 2025-11-25

**Authors:** Tassiane Cristine Santos de Paula, Cecília Menezes Farinasso, Mariana Millan Fachi, Haliton Alves Oliveira, Rosa Camila Lucchetta, Layssa Andrade Oliveira

**Keywords:** tomografia por emissão de pósitrons combinada à tomografia computadorizada, câncer de esôfago, estadiamento do câncer, sensibilidade e especificidade

## Abstract

**Introdução:**

O câncer de esôfago, uma das principais causas de morte no mundo, apresenta dois subtipos principais: o carcinoma de células escamosas e o adenocarcinoma. O diagnóstico precoce e o estadiamento correto são fundamentais para melhorar as taxas de sobrevivência. O estadiamento do câncer de esôfago pode ser realizado por exames de imagem como tomografia computadorizada (TC) e PET-CT (Tomografia por Emissão de Pósitrons combinada à Tomografia Computadorizada). No entanto, atualmente, o Sistema Único de Saúde (SUS) dispões apenas da TC para diagnóstico e estadiamento deste câncer. Dessa forma, este estudo visou avaliar a sensibilidade, a especificidade e a segurança da PET-CT) para o estadiamento tumoral comparada à TC.

**Método:**

Foi conduzida uma revisão sistemática com metanálise direta, incluindo estudos observacionais comparativos de acurácia diagnóstica e/ou de mudança de conduta clínica, envolvendo PET-CT e TC, tendo como padrão de referência exames histopatológicos e/ou cirurgia. As buscas foram realizadas em PubMed, Embase e Cochrane Library. O risco de viés foi avaliado pelas ferramentas QUADAS-2 e QUADAS-C, enquanto a qualidade da evidência foi analisada pelo sistema GRADE ( ) para estudos de acurácia.

**Resultados:**

Foram incluídos 15 estudos observacionais, oito avaliando a acurácia diagnóstica da PET-CT e da TC em comparação ao padrão de referência, e oito estudos avaliando a mudança de conduta clínica após a realização da PET-CT. Na detecção de linfonodos regionais, a PET-CT apresentou sensibilidade e especificidade de 66% (IC 95%: 57%-74%) e 60% (IC 95%: 53%-67%), respectivamente, enquanto para a detecção de metástases a distância foi de 88% (IC 95%: 78%-95%) e 91% (IC 95%: 87%-94%), respectivamente. Ainda, para a TC, a sensibilidade e especificidade para a detecção de linfonodos regionais foram de 57% (47%-66%) e 55% (48%-62%), enquanto para metástase a distância foram de 78% (52%-94%) e 77% (64%-87%), respectivamente. A PET-CT influenciou o manejo clínico, resultando em mudanças terapêuticas em 26% (IC 95% 24%-29%) dos pacientes ao reduzir a necessidade de cirurgias.

**Conclusão:**

A PET-CT pode apresentar sensibilidade e especificidade maior que a TC, tanto na detecção de linfonodos regionais quanto de metástase a distância. Além disso, a PET-CT demonstrou impacto na conduta clínica, influenciando as decisões terapêuticas e alterando a intenção de tratamento, contribuindo assim para a redução de intervenções cirúrgicas. Sendo assim, apesar dos possíveis efeitos indesejáveis associados à PET-CT, como reações alérgicas ao contraste, exposição à radiação ionizante, desconforto durante o exame e a possibilidade de resultados incorretos, como falsos-positivo ou negativos, a PET-CT ainda pode apresentar vantagens na detecção de linfonodos e metástase e manejo do tratamento quando comparado à TC.

